# Simple Quantification of Pentosidine in Human Urine and Plasma by High-Performance Liquid Chromatography

**DOI:** 10.1155/2017/1389807

**Published:** 2017-10-18

**Authors:** Ji Sang Lee, Yoon-Sok Chung, Sun Young Chang, Yi-Sook Jung, So Hee Kim

**Affiliations:** ^1^College of Pharmacy and Research, Institute of Pharmaceutical Science and Technology, Ajou University, Suwon 16499, Republic of Korea; ^2^Department of Endocrinology and Metabolism, Ajou University School of Medicine, Suwon 16499, Republic of Korea

## Abstract

Pentosidine is an advanced glycation end-product (AGE) and fluorescent cross-link compound. A simple high-performance liquid chromatographic (HPLC) method was developed for the detection and quantification of pentosidine in human urine and plasma. The mobile phase used a gradient system to improve separation of pentosidine from endogenous peaks, and chromatograms were monitored by fluorescent detector set at excitation and emission wavelengths of 328 and 378 nm, respectively. The retention time for pentosidine was 24.3 min and the lower limits of quantification (LLOQ) in human urine and plasma were 1 nM. The intraday assay precisions (coefficients of variation) were generally low and found to be in the range of 5.19–7.49% and 4.96–8.78% for human urine and plasma, respectively. The corresponding values of the interday assay precisions were 9.45% and 4.27%. Accuracies (relative errors) ranged from 87.9% to 115%. Pentosidine was stable in a range of pH solutions, human urine, and plasma. In summary, this HPLC method can be applied in future preclinical and clinical evaluation of pentosidine in the diabetic patients.

## 1. Introduction

**Figure 1:**
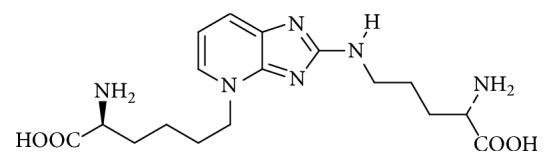
Chemical structure of pentosidine.

Pentosidine is an advanced glycation end-product (AGE) and a fluorescent compound (Figure 1). It is prepared from arginine, lysine, and pentose through sequential glycosylation and oxidation reactions [1]. In addition, pentosidine forms cross-links between lysine and arginine residues in collagen.

Pentosidine levels have been found to be elevated in end-stage renal disease, diabetes, and rheumatoid arthritis [2]. In patients with type 2 diabetes mellitus (T2DM) in particular, pentosidine level correlates with the presence and severity of diabetic complications [3]. Recently, pentosidine was studied as a potential diagnostic maker for these diseases. Pentosidine levels are elevated in the urine and serum of T2DM patients, with significant elevation first observed in the tissues of T2DM patients [4, 5]. Researchers studying diabetes have thus targeted pentosidine as a biomarker by measuring cross-linked collagen within various tissues [6]. A recent report also demonstrated that there is a strong correlation between nonenzymatic cross-linking of pentosidine in bone and plasma homocysteine. Both these parameters are known to provoke incident bone fractures, regardless of age and bone mineral density [7].

These studies did, however, face difficulties in measuring pentosidine levels in biological samples such as urine and plasma. Most analytical methods of high-performance lipid chromatography (HPLC) employ hydrolysis with concentrated HCl by heating at 110°C overnight in a sealed glass tube. This approach takes a long time and poses serious danger due to the strong acidity [2, 8, 9]. An additional method is therefore needed to remove the endogenous waste material and extract pentosidine, which involves complicated HPLC instrumentation such as a column-switching valve [10]. In the case of enzyme-linked immunosorbent assay (ELISA), the lower limit of quantification (LLOQ) for pentosidine was reasonable (0.1 pM), but this method requires enzyme digestion for 24 h and is less reproducible than the HPLC method [11].

To overcome these shortcomings, we developed a simple, fast, and accurate method to analyze pentosidine levels in human urine and plasma. This paper describes a simple sample preparation procedure (deproteinization) followed by an HPLC method in combination with fluorescence detection.

## 2. Experimental

### 2.1. Chemicals

Pentosidine was supplied by the Cayman Chemical Company (Ann Arbor, MI, USA). Heptafluorobutyric acid (HFBA) was obtained from Sigma Aldrich (St. Louis, MO, USA). Heparin and a 0.9% NaCl-injectable solution were obtained from JW Pharmaceutical Corporation (Seoul, Republic of Korea). Human plasma was purchased from BioChemed Services (Winchester, VA, USA). Various buffer solutions of pH 2.0, 4.01, 7.0, 9.21, and 10.0 were purchased from Mettler Toledo (Columbus, OH, USA). Other chemicals were of reagent grade or HPLC grade and were used without further purification.

### 2.2. HPLC Equipment

The chromatographic equipment used was a Shimazu Prominence LC-20A HPLC system (Shimazu, Kyoto, Japan) equipped with a low-pressure gradient unit (LC-20A), an autopurge, an in-line vacuum degassing autosampler (SIL-20A) with programmable temperature control, a heated column compartment, and a highly sensitive fluorescence detector (RF-20A/RF-20Axs) [12]. All components of the HPLC system were controllable through a CBM-20A system controller. Chromatographic separation of pentosidine was performed on a C_18_ reverse-phase column (AegisPak, 4.6 mm i.d., 25 cm *l*, particle size 5 *μ*m, Young Jin Biochrom, Seongnam, Republic of Korea) after sample filtration through a 0.45 *μ*m filter (Millipore, Billerica, MA, USA).

### 2.3. Chromatographic Conditions

The mobile phase used a binary gradient system at a flow rate of 0.8 ml/min. Solvent A was 100% acetonitrile and solvent B was 0.1% HFBA in water. The mobile phase was started at 90% solvent B. After 3 min, solvent B was changed using a linear gradient of 90–78% from 3 to 24 min, followed by a gradient of 78–5% from 24 to 33 min. After 33 min, solvent B was increased from 5% to 90% for 3 min. To equilibrate the column, 90% solvent B was maintained for another 3 min. Chromatograms were monitored with a fluorescence detector set at excitation and emission wavelengths of 328 and 378 nm, respectively. The sample injection volume was 100 *μ*l.

### 2.4. Preparation of Sample Standards in Biological Fluids

A stock solution of pentosidine was dissolved in dimethylsulfoxide (DMSO) at a concentration of 2 mM. Appropriate dilutions of the stock solution were made with HPLC-grade water. Standard solutions of pentosidine in human urine and plasma were prepared by spiking with the appropriate volume of the pentosidine stock solution to give final concentrations of 1, 2, 3, 4, 5, 10, 20, and 50 nM in human urine and 1, 2, 5, 10, 20, 30, 40, and 50 nM in human plasma. All the samples were stored in a −70°C freezer until HPLC analysis of pentosidine.

### 2.5. Biological Sample Preparation

Human urine was obtained from healthy male subjects. Plasma and urine samples were obtained from patients who visited the Endocrinology and Metabolism Unit of Ajou University Hospital, Suwon, Republic of Korea. The protocol and consent forms were approved by the Institutional Review Board of Ajou University School of Medicine (AJIRB-MED-SMP-14-109). The pentosidine detection assay followed an initial sample deproteinization method. Briefly, 200 *μ*l of acetonitrile was added to a 100 *μ*l aliquot of urine or plasma and mixed on a vortex mixer. After vortex-mixing, the mixture was centrifuged at 12,000 rpm for 10 min, and 250 *μ*l of the supernatant was transferred to a clean tube and evaporated under a gentle stream of nitrogen gas at 40°C (Eyela, Tokyo, Japan). The resultant residue was reconstituted with 150 *μ*l of 0.1 M HFBA and 100 *μ*l was injected onto the HPLC system.

### 2.6. Method Validation

#### 2.6.1. Selectivity

Basal endogenous pentosidine levels were first measured in nonpentosidine spiked urine and plasma samples. Urine and plasma samples were subsequently spiked with pentosidine and each nonspiked and spiked sample was assayed in triplicate in one run. Accuracy of the assay was assessed by measuring pentosidine levels in the spiked samples and calculating the proportion of the additional measured pentosidine levels [13].

#### 2.6.2. Linearity

The linearity of the relationship between the detector response and pentosidine concentrations was confirmed within the concentration range of 1–50 nM for both human urine and plasma samples. Calibration curves of the slope, intercept, and determination coefficients were calculated by plotting the peak area (*y*) for pentosidine versus the nominal concentrations (*x*) in standard human urine and plasma, using 1/*x*^2^ weighted least-square linear regression [12, 14].

#### 2.6.3. Recovery

The efficiency of the pentosidine analysis of human urine and plasma was determined by comparing the mean responses, from three replicate standard samples for each pentosidine concentration, in the human urine and plasma, with the mean response of pentosidine from aqueous standards at equivalent concentrations following the deproteinization process. Recovery of pentosidine was determined at all concentration ranges studied in human urine and plasma.

#### 2.6.4. Precision and Accuracy

To evaluate the precision and accuracy of the method, we analyzed the standard samples at eight concentration levels (lowest, 1 nM; highest, 50 nM) for urine and plasma, in three replicates on three separate days. The response factor for each standard sample was calculated by subtracting the nonspiked response factor from the spiked standard response factor, followed by dividing by the spiked concentration. The mean standard deviation (SD) and the ratio of SD to the mean coefficient of variation (CV) were calculated and used to evaluate the precision of the assay. The accuracy of the assay was assessed by comparing the calculated mean concentrations to the actual concentrations of serial dilutions. Accuracy was required to be within 85–115% of the nominal concentration, and the intra- and interday precisions (represented by CV) were not to exceed 10% [12, 14].

#### 2.6.5. Lower Limits of Detection and Quantification

The lower limit of detection (LLOD) was defined as the peak signal of pentosidine equal to three times the average background noise level. The LLOQ was defined as the lowest concentration of pentosidine giving a linear relationship between the spiked standard response factor/nonspiked response factor and the spiked concentration, with an accuracy of 100 ± 15% [13].

### 2.7. Stability Test

The stability of pentosidine in the biological samples was assessed by comparing the final concentration with the initial concentration of pentosidine following incubation under the designated conditions. The final data were expressed as a percentage of the initial concentration.

#### 2.7.1. Short- and Long-Term Stability

The stability of pentosidine in human urine and plasma was evaluated by analyzing triplicates of the samples exposed to different conditions (incubation time and temperature), at concentrations of 5 and 50 nM. Short-term stability in human urine and plasma was assessed by analyzing samples kept at 4, 25, and 37°C for 1, 2, 3, 4, 6, 8, 12, and 24 h. Long-term stability was determined by assaying samples after storage at −70°C for 1, 2, 4, and 7 days, as well as 2, 4, 6, and 8 weeks. The final results were compared with those obtained for freshly prepared samples. All samples were kept at −70°C freezer until the HPLC analysis of pentosidine.

#### 2.7.2. Stability in Buffered Solutions at Various pH

Pentosidine stock solution was spiked in each glass test tube containing 5 ml of a buffered solution at pH 2.0, 4.01, 7.0, 9.21, and 10.0 to final pentosidine concentrations of 5 and 50 nM. After vortex-mixing, each test tube was placed in a water-bath shaker at 25 and 37°C for 1, 2, 3, 4, 6, 8, 12, and 24 h. After incubation, a 50 *μ*l aliquot of the buffer solution was taken from each test tube at the designated time points and injected onto the HPLC column.

## 3. Results and Discussion

**Figure 2:**
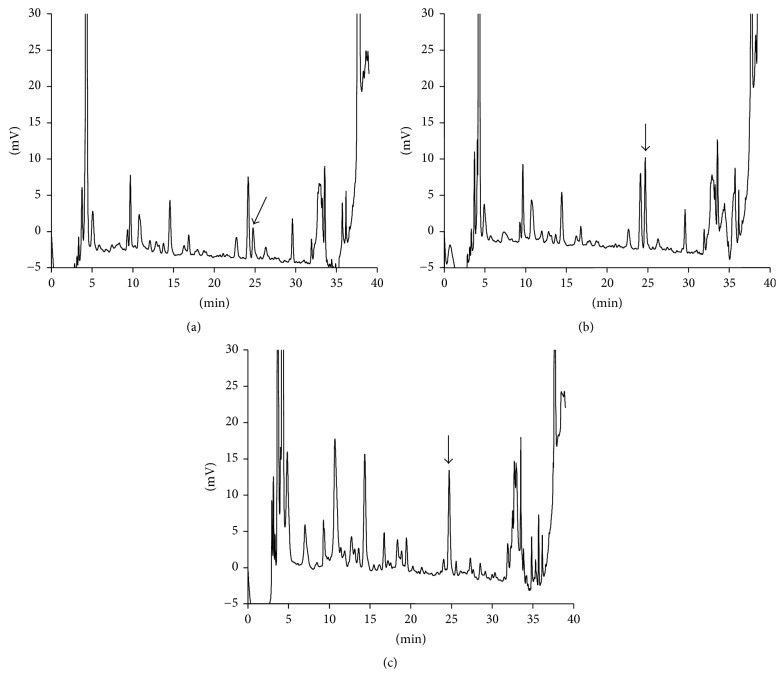
Chromatograms after deproteinization of a human urine blank (0.663 nM) (a), urine standard spiked with 1 nM of pentosidine (b), and type 2 diabetes mellitus (T2DM) patient's urine (2.28 nM) (c). The arrows show the pentosidine peak. The human urine used in (a) and (b) was obtained from a healthy male subject.

**Figure 3:**
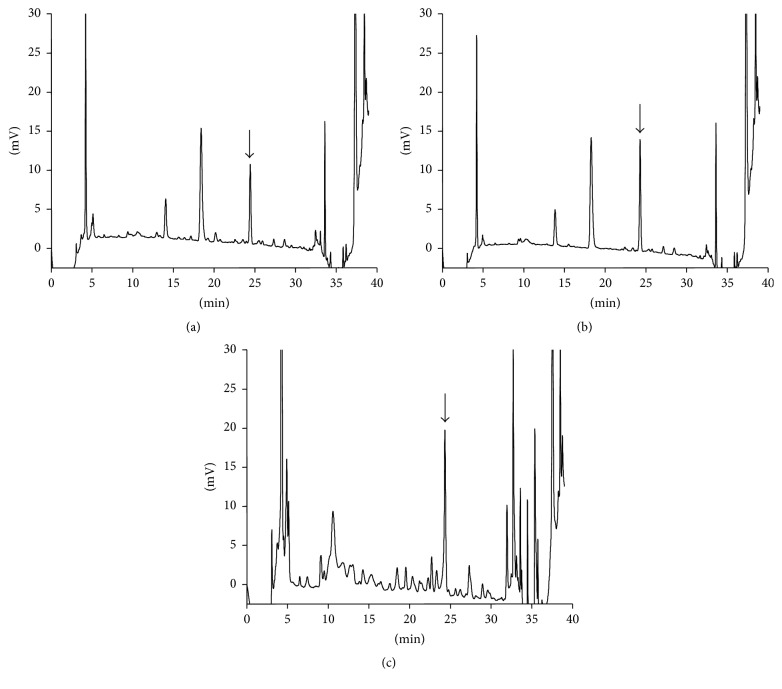
Chromatograms after deproteinization of a human plasma blank (5.60 nM) (a), plasma standard spiked with 1 nM of pentosidine (b), and a type 2 diabetes mellitus (T2DM) patient's plasma (13.9 nM) (c). The arrows show the pentosidine peak. The human plasma used in (a) and (b) was purchased from BioChemed Services.

Based on fluorescence absorption spectra, the optimal excitation and emission wavelengths appeared at 328 and 378 nm, respectively. These wavelengths were therefore used for all HPLC analyses of pentosidine, taking into consideration the background fluorescence and assay sensitivity [15].  Figure 2 shows typical chromatograms of blank human urine (Figure 2(a)), human urine spiked with 5 nM pentosidine (Figure 2(b)), and urine from a T2DM patient (Figure 2(c)); the corresponding chromatograms for human plasma are shown in Figures 3(a), 3(b), and 3(c), respectively. The pentosidine peak was found to be symmetrical and eluted at approximately 24.3 min. There was no interference from other endogenous substances in any of the biological samples, except basal endogenous pentosidine.

**Figure 4:**
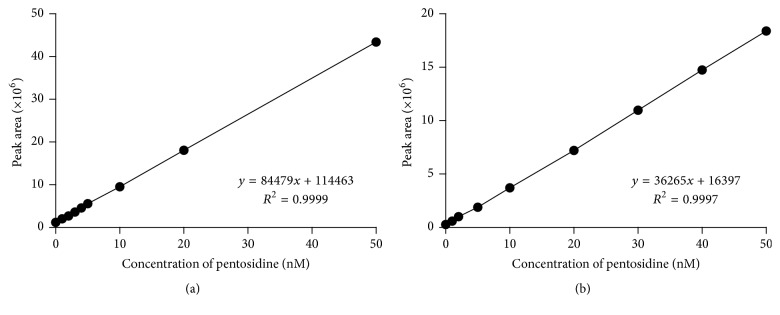
Linear regression and correlation coefficients (*R*) of pentosidine at various concentrations in human urine (a) and plasma (b).

The correlation coefficients (*R*) of the standard curves for pentosidine in human urine and plasma samples were greater than 0.999 and 0.980, respectively (Figures 4(a) and 4(b)) The LLOQ for pentosidine in human urine and plasma was 1 nM based on a linear relationship between the response factor, subtracting the nonspiked response factor from the spiked standard response factor, and the spiked pentosidine concentration with an accuracy of 100 ± 15% (Tables 1 and 2).

Precision was defined as the CVs between three replicate samples. The mean intraday CVs of pentosidine in human urine (Table 1) and plasma (Table 2) were generally low, 6.34 (5.19–7.49%) and 6.80% (4.96–8.78%), respectively. The mean interday CV values of the same samples across three consecutive days were 9.45 and 4.27% in human urine and plasma, respectively. The mean accuracy of pentosidine was 89.5–106% and 87.9–103% for human urine and plasma, respectively (Tables 1 and 2). The precision and accuracy values were well within the acceptable ranges, as described by the United States Food and Drug Administration [16]. The mean recoveries of pentosidine in human urine and plasma were 109% (97.2–114%) and 52.1% (41.6–57.3%), respectively (Tables 1 and 2). The reason for the low recovery of pentosidine from human plasma might be the higher pH in plasma than in urine [17]; the average pH is 5.5 and 7.4 in human urine and plasma, respectively. Pentosidine is less soluble in higher pH solutions and is not completely recovered from collagen in plasma [17].

Pentosidine was stable in human urine at 4, 25, and 37°C; more than 91.5% of the compound remained at the original concentrations of 5 and 50 nM after 24 h incubation (data not shown). Pentosidine was also stable in human plasma at both 5 and 50 nM with up to 24 h of incubation; more than 92.0% of pentosidine was recovered following incubation at 4, 25, and 37°C (data not shown). Pentosidine was also stable in the buffer solutions of pH 2.0, 4.01, 7.0, 9.21, and 10.0; the recoveries of the spiked pentosidine (5 and 50 nM) after 24 h of incubation ranged from 90.3 to 109% at 25 and 37°C, respectively (data not shown). We also found that pentosidine was stable at −70°C for long periods of time; more than 99.0% and 94.0% of pentosidine remained for up to 8 weeks in human urine and plasma, respectively, at concentrations of both 5 and 50 nM (data not shown).

Pentosidine level was found to be elevated in both urine and serum in T2DM patients [3–5]. We therefore applied the developed HPLC method to our T2DM patients' urine and plasma to evaluate the pentosidine levels and confirmed the detection of pentosidine in both patients' urine and plasma samples in T2DM patients. Based on the results of our study, our newly developed HPLC method can be applied to detect pentosidine in both human urine and plasma samples as a diagnostic marker in the diabetic patients.

## 4. Conclusions

In summary, a simple and sensitive HPLC method was developed for the determination of pentosidine levels in human urine and plasma and validated based on USA Food and Drug Administration guidelines. In addition, pentosidine was found to be stable in human urine, plasma, and various buffer solutions ranging from pH 2 to 10 for up to 24 h. These results support the use of pentosidine in clinical tests for diabetes.

## Figures and Tables

**Table 1:** Mean response factors, coefficients of variation (CV), accuracy, and recovery of pentosidine at various concentrations in human urine.

Concentration (nM)	Response factor ± SD (% CV)	Accuracy^a^ (%)	Recovery^b^ (%)
1	81986 ± 7746 (9.45)	99.6	112
2	73662 ± 884 (1.20)	89.5	97.2
3	80179 ± 3599 (4.49)	97.4	109
4	83676 ± 5233 (6.25)	102	111
5	87057 ± 1527 (1.75)	106	114
10	83147 ± 1457 (1.75)	101	112
20	84339 ± 2234 (2.65)	102	112
50	84359 ± 1510 (1.79)	103	105

Measurements were conducted three times in separate days; the response factor was calculated by subtracting nonspiked response factor from the spiked standard response factor followed by dividing by the spiked concentration; data are expressed as mean response factor ± standard deviation (SD) (*n* = 3); values in bracket are intraday coefficients of variation (CV). ^a^(Mean  measured  concentration/spiked  concentration) × 100; ^b^Relative recovery compared with water.

**Table 2:** Mean response factors, coefficients of variation (CV), accuracy, and recovery of pentosidine at various concentrations in human plasma.

Concentration (nM)	Response factor ± SD (% CV)	Accuracy^a^ (%)	Recovery^b^ (%)
1	31503 ± 719 (2.28)	87.9	41.6
2	36813 ± 104 (0.28)	103	54.0
5	36456 ± 1154 (3.16)	102	51.4
10	36417 ± 673 (1.85)	102	55.7
20	35682 ± 1242 (3.48)	99.6	57.3
30	36374 ± 682 (1.88)	102	53.0
40	36677 ± 461 (1.26)	102	53.6
50	36638 ± 1737 (4.74)	102	50.5

Measurements were conducted three times in separate days; The response factor was calculated by subtracting nonspiked response factor from the spiked standard response factor followed by dividing by the spiked concentration; data are expressed as mean response factor ± standard deviation (SD) (*n* = 3); values in bracket are intraday coefficients of variation (CV). ^a^(Mean  measured  concentration/spiked  concentration) × 100; ^b^Relative recovery compared with water.
